# Persistent CO_2_ Reduction Performance of an Ag Nanoparticle Gas Diffusion Electrode in Realistic Dynamic PV-Driven Operation

**DOI:** 10.1021/acs.energyfuels.5c03523

**Published:** 2025-09-24

**Authors:** Thérèse Cibaka, Tsvetelina Merdzhanova, Oleksandr Astakhov, Sergey Shcherbachenko, Guangxin Liu, Chuyen van Pham, Uwe Rau, Peter Strasser

**Affiliations:** † Forschungszentrum Jülich GmbH, IMD-3 Photovoltaik, Jülich 52428, Germany; ‡ Helmholtz-Institute Erlangen-Nürnberg for Renewable Energy (IET-2), Forschungszentrum Jülich, Cauerstr. 1, Erlangen 91058, Germany; § Jülich Aachen Research Alliance (JARA-Energy) and Faculty of Electrical Engineering and Information Technology, RWTH Aachen University, Aachen 52062, Germany; ∥ Technical University of Berlin, Institute of Chemistry, Berlin 10623, Germany

## Abstract

Progress in the development of CO_2_ reduction catalysts has revealed more stable and selective options for solar fuel production. In most cases, the catalysts are tested under steady-state conditions. However, to become a reliable long-term storage solution for renewable energy, particularly photovoltaics (PV), CO_2_ electroreduction must tolerate power intermittency. Direct coupling of CO_2_ electrolyzers to PV devices enables carbon utilization and efficient energy storage but requires catalysts that maintain consistent performance under dynamic power input. Herein, we select an Ag nanoparticle gas diffusion cathode with stable CO production across a wide current density range. The system, directly coupled to a hardware-emulated Si-PV module operating under a realistic sunny day profile, achieves 96% energy coupling efficiency and reaches a cumulative solar-to-chemical (CO) efficiency of 8.8% in 1 day. This study demonstrates the potential of Ag-based cathodes for robust performance in variable PV-powered systems and introduces a novel test methodology that better reflects real-world PV-electrolyzer integration, thereby advancing practical implementation of solar-driven CO_2_ reduction.

## Graphical abstract

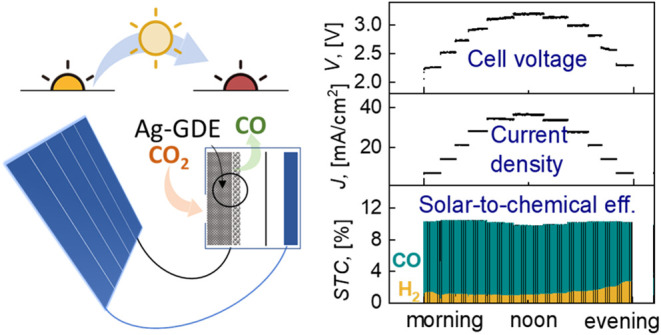

## 1 Introduction

Two major renewable energy sources, wind and solar, constitute 63% of the global renewable power capacity.^1^ More specifically, the solar market is growing at a faster rate than initially predicted, thereby making a significant contribution to the green energy transition.^2^ However, the efficient use of photovoltaic (PV) capacity is limited by the temporal mismatch between generation and demand, while power intermittency hinders grid compatibility. The optimal use of PV energy requires an energy storage strategy, particularly on a long timescale, to address seasonality. An ideal storage solution effectively absorbs intermittent DC power from PV generation while maintaining consistent performance across a broad dynamic range. In view of multiterawatt-scale PV generation, any PV-storage solution must utilize abundant materials and up-scalable synthesis methods.

The conversion of rampant CO_2_ into fuels or valuable chemicals through the electrochemical CO_2_ reduction reaction (CO_2_RR) presents a high-potential strategy for energy storage and carbon utilization when powered by renewable energy, such as PV. For this purpose, catalysts used in electrochemical cells (ECs) must handle intermittent power input and industrially relevant current densities (>200 mA/cm^2^) while maintaining high selectivity toward target products.^3^ The realistic intermittent operating context of PV storage has been largely neglected in catalyst development and testing. The reported catalysts often have a narrow window of operating voltages with high faradaic efficiency toward the target product.^4,5^

Our previous works have demonstrated the self-sustained operation and high performance of PV coupled with batteries,^6,7^ ECs,^8,9^ and their combinations.^10,11^ Connecting PV and EC systems without full integration allows independent optimization and preserves device integrity. Direct coupling also enables the on-site use of PV-generated DC power for solar fuel production, thereby alleviating grid congestion. At the laboratory scale, solar-to-chemical efficiency (*STC*) or the ratio of solar energy irradiated on the exposed area of PV converted into chemicals by EC is the highest for direct-coupled PV-EC devices.^12–18^ The high performance of these systems is based on matching the PV and EC current voltage characteristics in such a way that their intersection, the operating point (OP) of the PV-EC device, is in a close vicinity to the maximum power point (MPP) of the PV device. The proximity of the OP to the MPP or the degree of power coupling is quantified using the power coupling factor (or power coupling efficiency).^19–21^ In the case of intermittent power input, the power coupling factor, which represents a specific moment in time, is not always an adequate metric for evaluating the coupling performance over a period of operation. Therefore, it is useful to evaluate the energy coupling factor, which is the ratio of the energy utilized by the EC to the maximum energy that the PV device could deliver over the same period.

CO_2_ reduction catalysts in directly coupled PV-EC systems are typically tested under fixed lab conditions of PV (1 sun, AM 1.5G, 25 °C),^8,9,12–18,22^ where the optimization of PV-EC power coupling is straightforward. In reality, the PV current–voltage (*I–V*) output characteristics vary with the irradiance (G) spectrum and module temperature (T),^23–27^ shifting both the operating and maximum power points. This has two major implications: the variation in PV-EC power coupling efficiency and the variation in the catalyst behavior in terms of selectivity and potential stability. In our work, we address both aspects. We studied the behavior of a silver catalyst optimized for a broad dynamic range of operating conditions in a PV-EC system, optimized for high power and energy coupling efficiency in dynamic operation.

Silver is a relatively abundant catalyst with high CO selectivity, making it a strong candidate for large-scale CO_2_RRs. This work employs an in-house developed Ag nanoparticle gas diffusion electrode (Ag-GDE)^28^ for testing as a CO-selective cathode with a variable PV power input. The Ag gas diffusion electrode (GDE) was fabricated via a scalable industrial method, doctor blading, of custom silver ink onto a gas diffusion layer. The ink was optimized for uniform coating and achieved approximately 94% CO selectivity across 50–200 mA/cm^2^.^29^ The Ag-GDE cathode, the focus of our study, was tested in a full-flow CO_2_RR electrochemical cell using IrO*_x_* as the benchmark anode due to its high activity and low overpotential. However, for practical implementation, more affordable and earth-abundant alternatives are needed. While the oxygen evolution reaction is sluggish, the ethylene glycol oxidation reaction driven by noble-metal-free catalysts like NiFe LDH offers lower oxidation potentials at the anode,^30^ thereby increasing the overall cell voltage efficiency and *STC*_CO_. In this study, we selected a well-established reference anode material (IrOx) and reaction (OER) as reference points to maintain focus on the Ag-GDE and facilitate a comparison with previously reported work.

To study the interplay of the electric characteristics of PV and EC devices under realistic but reproducible and well-controlled conditions, we employed a novel high-precision PV emulation device dedicated to this type of study.^31^ Via the emulation of PV IVs, any realistic irradiance and temperature scenarios for PV output can be tested with high reproducibility. The output of a state-of-the-art silicon heterojunction (SHJ) or Si–PV module with 23.8% efficiency (1 sun AM 1.5G and 25 °C) was emulated for a typical sunny day (sunrise to sunset) in Golden (Colorado, USA) in a stepwise profile for irradiance from 0.2 to 1.1 to 0.2 sun and temperatures from 20 to 53.5 °C. The same “day” was repeated three times in the experiment. The PV-EC device performance was assessed via the power and energy coupling factor, while the Ag-GDE was evaluated for product selectivity, stability, and solar-to-chemical efficiency. The results highlight the strong potential of the Ag-GDE for efficient CO_2_ reduction and solar energy storage in direct-coupled PV-EC systems.

## 2 Results and Discussion

### 2.1 Realistic Irradiance, Temperature Profile, and PV-EC Current–Voltage Electric Interplay

For ambient conditions and test scenarios, we relied on the widely used NREL data set for PV modules operating in Golden, Colorado (USA).^32^ To maximize PV energy utilization, the PV-EC power coupling efficiency must be optimized to the irradiance (*G*) and temperature (*T*) ranges corresponding to the maximum energy yield of the specific PV installation. Figure 1a shows the cumulative yearly energy output of an SHJ PV module in Golden, Colorado, as a function of *G* and *T*. The energy peaks at 1 sun and 49 °C. The yellow circles mark 11 representative *G*–*T* pairs simulating a typical high-output day, forming a time series from 0.2 sun–20 °C to 1.1 sun–53.5 °C and back, condensed into a 5-h profile (Figure 1b). The morning and afternoon irradiance profiles are symmetric, peaking at 1.1 sun, with higher afternoon temperatures due to ambient warming. Figure 1c displays the PV IV curves of an SHJ module emulated in this work under these 11 *G*–*T* conditions (Table M1 in the Method Section-Supporting Information), scaled to match the EC polarization curve presented in blue. As the irradiance increases, the PV current at the maximum power point (*I*_MPP_) also increases.

**Figure 1:**
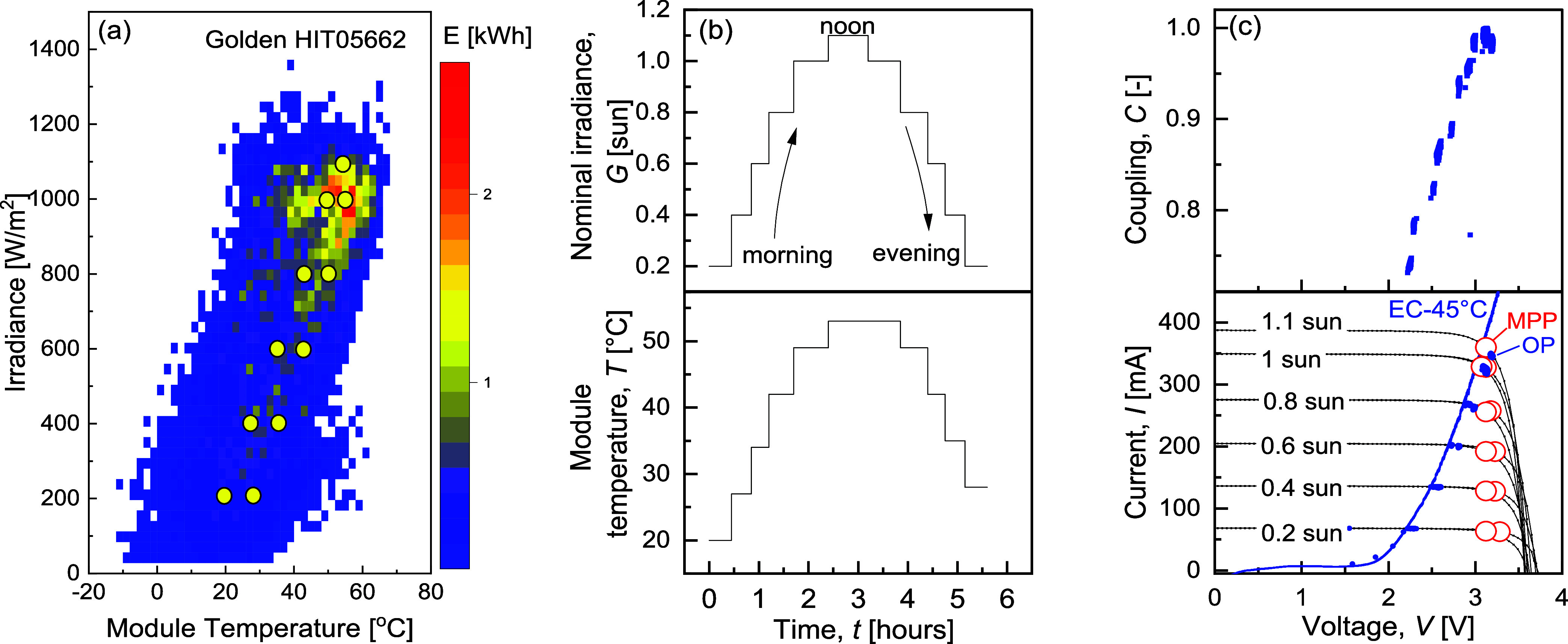
(a) Maximum yearly cumulative energy of an SHJ PV module in Golden plotted against irradiance and temperature conditions. The yellow circles indicate 11 representative irradiance and temperature combinations of a summer day. (b) Eleven dynamic nominal irradiance and module temperature conditions simulating a single summer day condensed in 5 h. (c) Bottom section: EC polarization curve crossing PV–*I–V* characteristics under the 11 representative *G* and *T* conditions. The PV-EC operating points are recorded as blue points on the PV–*I–V* curve, and MPPs are represented as red hollow circles. Upper section: PV-EC power coupling at each *V*_OP_ during a single day’s operation.

At equal irradiances but higher temperatures (afternoon), the current–voltage curves shift to lower voltages, with *V*_MPP_ decreasing, as is typical for most solar cells.^27^ Separate effects of temperature and irradiance on PV *I–V*s are available in Supporting Information (Figure S1). The current output of PV increases with irradiance from 67 mA at 0.2 sun to 360 mA at 1.1 sun, while the EC operating voltage increased from 2.23 to 3.2 V, as presented in Figure 1c.

**Figure 2:**
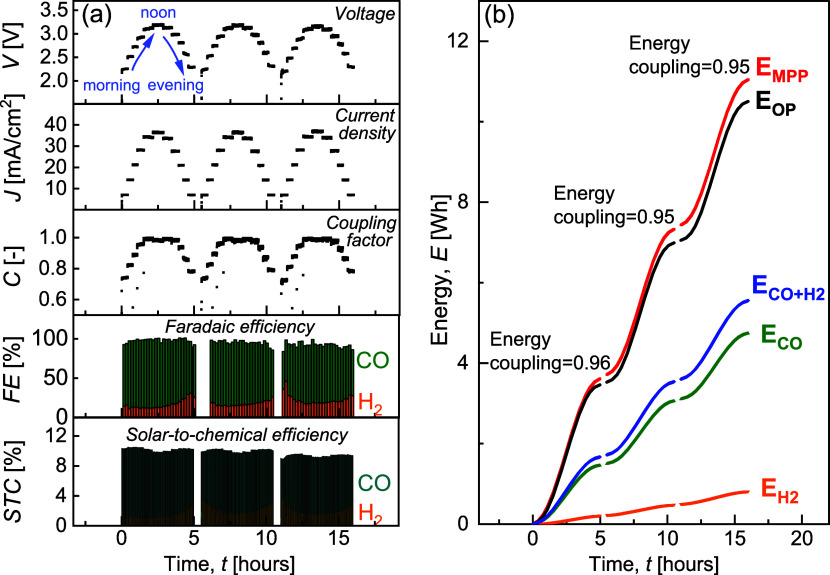
(a) Temporal evolution of PV-EC operating voltage and current density, power coupling factor of PV-EC, and faradaic efficiencies at operating points during a simulated accelerated triple-day. (b) Cumulative energies involved in each step of the solar-to-chemical conversion efficiency during the triple-day and daily energy coupling.

In order to achieve optimal PV-EC power coupling under peak irradiance conditions (1.1 sun, 53.5 °C), the PV characteristics in Figure 1c are scaled for the PV emulator to reproduce the IVs of a five-cell serially connected PV module with a PV-to-EC area ratio of 4.7. PV-EC scaling defines the device’s operating power range and is critical for techno-economic feasibility. Higher PV-to-EC area ratios (A_PV_/A_EC_) are usually more economically favorable. One of the main contributors to EC cost is the electrode materials.^33^ The use of abundant materials is a determinant to insert PV-EC as a commercially competitive, sustainable, and resilient solution. The silver load of our cathode was 2 mg/cm^2^, which at an area ratio of 4.7 corresponds to the use of approximately 4.3 g of silver in EC per m^2^ of the Si-PV module to realize CO_2_ reduction storage. This is a significant amount compared to the Ag use in modern Si-PV panels, which ranges from approximately 0.3 to 0.5 g/m^2^.^34^ Further development, such as improving the electrochemical cell design to boost the current density at a low overpotential, is likely to increase the A_PV_/A_EC ratio_.

The PV-EC power coupling at each *V*_OP_ is shown in the upper part of Figure 1c. The power coupling in the system remains above 0.70 at all *V*_OP_, peaking at 0.98–1 at the highest irradiances, where coupling losses can be most critical.

### 2.2 Performance of the Ag-GDE in PV-EC under Realistic Triple Sunny Day Conditions

The Ag-GDE performance under variable power input was evaluated via PV-EC operation over three identical 5-h simulated days, separated by 30 min night phases, compressing the full experiment into a 16 h period. The temporal evolution of PV-EC operation over 13 h of nonaccelerated daytime and 20 min of highly accelerated daytime is presented in Supporting Information (Figures S2 and S3) and demonstrates that the system can withstand multiple daily fluctuations while maintaining a reproducible power coupling efficiency, stable current–voltage behavior, and consistent CO selectivity. In Figure 2a, we present the temporal evolution of the operating voltage and current density (*V*_OP_ and *J*_OP_) of the EC, PV-EC power coupling factor *C*, faradaic efficiency (*FE*) of the products, and solar-to-chemical efficiency (*STC*). Under day-and-night *G* and *T* cycling conditions, PV-EC maintained reproducible *V*_OP_ and *J*_OP_, and significant power coupling efficiency, demonstrating Ag-GDE stability over the course of 16 h operation with a fluctuating power input. SEM images of fresh and used Ag-GDEs (Figure S4, Supporting Information) show that, after extended operation, some Ag particles appear to have fused together, but no changes in the operating current density were observed. Further investigation is required to determine the cause and implications of this morphological change.

The available PV power output is primarily affected by irradiance. As a result, *V*_OP_ and *J*_OP_ in Figure 2a followed the irradiance pattern in Figure 1b. Throughout the simulated day, the PV maximum power (*P*_MPP_ = *V*_MPP_ × *I*_MPP_) ranged between 200 and 1200 mW, and our system maintained a power coupling factor between 0.73 (in the mornings) and 1 (around noon). This result is comparable to the power efficiency profiles of maximum power point trackers for a similar range of power,^35^ highlighting the relevance of direct coupling, particularly when operating across a broad range of power values.

In Figure 2a, the Ag-GDE exhibited a total *FE* (CO and H_2_) of approximately 100% on day 1, 97 and 94% on day 2 and day 3. The CO faradaic efficiency remained consistently higher than that of H_2_ throughout the simulated triple-day. Overall, Ag-GDE in our PV-EC system favored CO_2_RR over HER across the full EC current density range (7–37 mA/cm^2^), indicating the high potential of the Ag cathode for PV storage applications.

Up to the third hour of operation on day 1, and independent of *V*_OP_ and *J*_OP_ fluctuations, both *FE*_CO_ and *FE*_H2_ remained at 86 and 14%, respectively. In general, *FE*_H2_ typically increases at a low overall cell voltage.^4,36–38^ In this study, this refers to the morning and evening. However, on day 1, the cathode is highly CO-selective, and the increase in H_2_ evolution at a lower cell voltage in the morning is suppressed. Toward the evening, after 4 h of operation, when the cell voltage is lower again, the CO selectivity is somewhat reduced but still predominant.

We attribute this behavior to the use of a freshly prepared, highly hydrophobic Ag-GDE, which effectively inhibits the electrolyte (1M, KHCO_3_)–catalyst interaction. However, over time, the presence of ions such as OH^–^, HCO_3_^–^, and K^+^, and possible salt precipitation can decrease the hydrophobicity of the fresh Ag-GDE and presumably allow more electrolyte into the triple-phase boundary, increasing H_2_ formation as a result of water reduction.^39,40^ The decrease in hydrophobicity was confirmed by the presence of electrolyte-derived potassium structure in the cross section of Ag-GDE and by a reduction in the contact angle between a water droplet and Ag-GDE surface, decreasing from 146° for the fresh Ag-GDE to 133° after 1 day of operation, and further to 116° after 3 days. The corresponding contact angle measurements and EDX maps of the cross section of the used Ag-GDE are shown in Figures S5 and S6, respectively, in the Supporting Information.

At the beginning of day 2, the Faradaic efficiency toward CO was higher than that at the end of day 1, despite operating at a similar voltage range. Multiple parameters are likely to affect selectivity, such as the hydrophobicity of Ag-GDE, HCO_3_^–^ availability, structural changes in silver nanoparticles, insertion of K^+^ species in the surface and cross section of Ag-GDE, etc. We suggest that this improvement is related to the electrolyte refreshment carried out prior to day 2, which appears to enhance CO_2_ reduction selectivity. A similar trend can be observed in Figure S8 of the Supporting Information for the experiment conducted at a constant voltage of 3.0 V, where an electrolyte refreshment after 5 h led to improved CO selectivity. At the beginning of day 3, after a total of 10 h of operation, no improvement in CO selectivity was observed compared to the night of day 2, despite the electrolyte refreshment. Presumably, the effects of the refreshment were overpowered by another time-dependent effect, such as structural changes in the GDE.

On day 2 and predominantly on day 3, Ag-GDE showed higher CO selectivity at elevated *V*_OP_, coinciding with peak irradiance or “*near noon*” when PV-EC power and *C* were highest. The *STC* values remained at approximately 10% throughout the course of the triple-day experiment. *STC*_CO_ and *STC*_H2_ during the days followed the pattern of *FE*_CO_ and *FE*_H2_.

Figure 2b shows the PV-EC performance as cumulative energy over the “triple-day,” with reproducible energy coupling factors of 0.96, 0.95, and 0.95 for the three identical days. Power coupling captures the performance at a specific moment in time, whereas energy coupling represents the cumulative fraction of extractable PV energy delivered to the EC system over a given period. In practical applications with intermittent PV outputs, energy coupling serves as a more relevant metric over extended durations.

Table 1 presents a summary of the total solar-to-chemical efficiencies and *STC* toward CO and H_2_ after 1, 2, and 3 days. The *STC* decreased from 10.5% on day 1 to 10.2% on day 3, mainly due to a lower total faradaic efficiency of the collected products. Although CO remained dominant, its selectivity was the lowest on day 3. On days 1 and 2, the decrease in CO selectivity is counterbalanced by an increase in H_2_ selectivity. Day 1 was repeated in 3 independent experiments.

**Table 1:** Summary of Cumulative Solar-to-Chemical Efficiencies and *STC* toward CO and H_2_ after 1, 2, and 3 days

STC, %	STC H_2_, %	STC CO, %	days
10.50 ± 0.03	1.70 ± 0.01	8.80 ± 0.02	1 day
10.5	2.3	8.2	2 days
10.2	2.4	7.8	3 days

The STC_CO_ decay under constant voltage operation (*V*_OP_ of 3.0 V) is presented in the Supporting Information, Figures S8 and S9. For comparison, we ensure that in both cases, the same total charge was passed, 2359.6 mA·h. Under dynamic operations, the *STC*_CO_ values decreased from 8.8 to 8.2% from day 1 to day 2. Under constant voltage conditions, the system maintains STC_CO_ values between 8.3 and 8.4%, owing to the stable CO faradaic efficiency. On the first day under dynamic operation, PV-EC benefits from low *V*_OP_ at the beginning and end of the simulated day, with very high CO selectivity for most part of the day. However, on day 2, the overall CO selectivity decreased. Additionally, Figure S10 in the Supporting Information presents the static CO_2_RR performance at potentials ranging from 2.4 to 3.4 V, expressed in terms of faradaic efficiency. Whether powered by PV or by a source measurement unit at different potentials, the CO_2_RR consistently achieves a high CO selectivity.

The reported *STC*_CO_ efficiencies improved from 6 to 19% due to advances in CO_2_RR catalysts,^41–46^ EC design, and notably, PV efficiency. Comparing PV-EC systems^5,47–58^ requires accounting for PV performance, as shown in Figure 3, which plots *STC*_CO_ versus PV efficiency. High *STC*_CO_ values (13–20%) are typically achieved with high-efficiency PVs like GaInP/GaInAs/Ge or GaAs (PV efficiency above 28%) and are mostly used in concentrator photovoltaics (CPV).^52–57,59^ However, their cost, complexity, and limited performance under diffuse light hinder broad adoption. Silicon-based PV remains the dominant and scalable choice, making it the most practical option for coupling with CO_2_ electrolysis systems.

**Figure 3:**
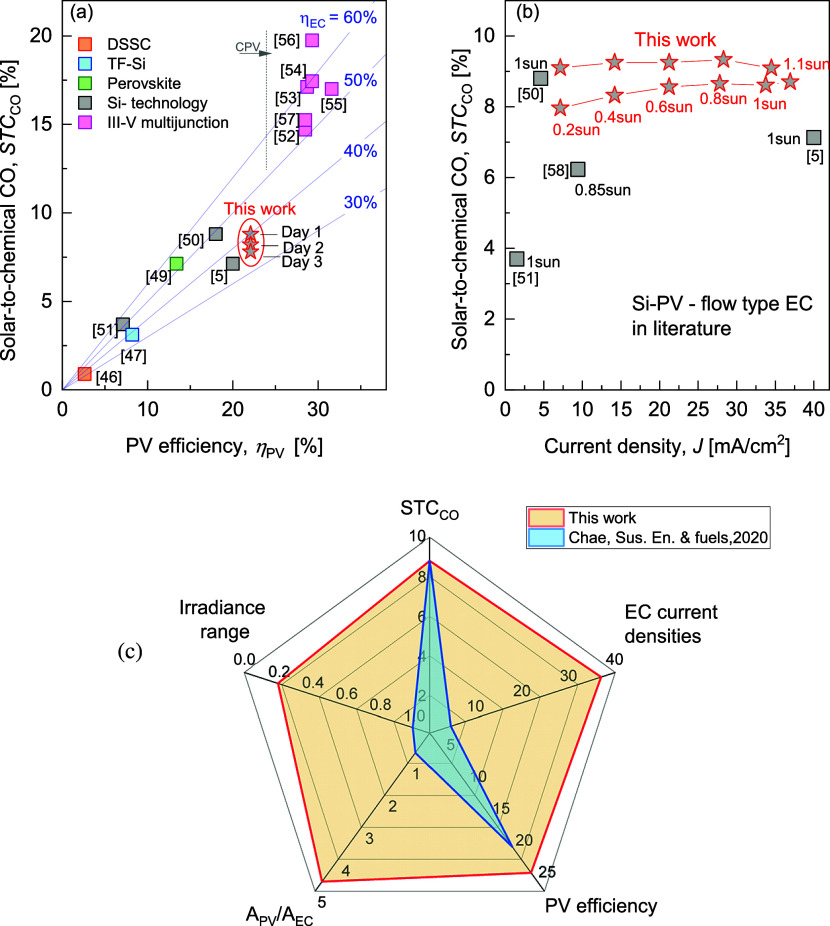
(a) Solar-to-chemical CO efficiency as a function of PV efficiency for different PV technologies. The orange square represents dye-sensitized solar cells (DSSC),^46^ The light blue square represents thin-film silicon technology (TF-Si),^47^ the green square represents perovskite technology,^49^ the gray squares represent Si-technology PV,^5,50,51^ and the violet squares represent III–V multijunction PV, used as CPV.^52–57^ The blue guidelines represent *STC*_CO_ evolution plotted against PV efficiency when η_EC_ values, EC efficiency toward CO, are 30, 40, 50 and 60% with a maximum PV-EC power coupling. (b) Solar-to-chemical CO efficiency as a function of current density for reported Si–PV connected to flow-type EC.^5,50,51,58^ (c) *STC*_CO_, current densities, PV efficiency, PV-to-EC area ratio, and tested irradiances for our Si–PV-EC compared to the best prior results from Chae et al.

Si–PV connected to CO_2_RR flow-type EC devices shows typical *STC*_CO_ between 2.5 and 8%.^5,50,51^ Herein, we report a notable cumulative *STC*_CO_ efficiency of 8.8% after day 1, 8.2 and 7.8% after day 2 and day 3, respectively, using silver nanoparticle GDE. Furthermore, in realistic scenarios, PV-EC systems operate across low to high current densities daily. A high-performance cathode should maintain a stable *STC*_CO_ over this range. In this study, Ag-GDE in flow-type EC directly connected to emulated Si-PV promotes high and stable *STC*_CO_ across a wide dynamic range of electrolyzer current densities, from 7 mA/cm^2^ to 37 mA/cm^2^, as illustrated in Figure 3b.

To date, the highest *STC*_CO_ (8.03%) in Si-PV-flow EC systems was reported by Chae et al. using a nanoporous silver film, where the CO_2_ supply was restricted to the fraction dissolved in the electrolyte. The system achieved a CO selectivity of 94% but was limited to low current densities (2–6 mA/cm^2^, tested at 4.6 mA/cm^2^) and was operated under constant standard conditions of 1 sun.^50^ In contrast, our cathode architecture incorporates a gas diffusion electrode that maximizes the interaction between CO_2_ molecules and the silver catalyst particles, enabling higher current densities (7–37 mA/cm^2^) with high CO faradaic efficiency.

The tested catalyst can be transferred to more industry-relevant advanced zero-gap membrane electrode assembly electrolyzers.^60–62^ The Ag-GDE and testing method enabled an accurate assessment of its viability for PV-powered CO_2_ reduction under realistic conditions, including dynamic *G* and *T* cycling, day–night transitions, a wide current density range, and a high PV-to-EC area ratio (4.7). Figure 3c illustrates the operational context and *STC*_CO_ of PV-EC (with silver nanoporous film cathode) from the best prior result by Chae et al.,^50^ compared with the broader investigation scope and *STC*_CO_ (on day 1) achieved in this work with Ag-GDE.

To the best of our knowledge, the cumulative *STC*_CO_ values after day 1 (8.8%) and day 2 (8.2%) are the highest reported for Si-PV-flow-type EC class devices for current densities above 4.6 mA/cm^2^.

## 3 Conclusions

In summary, although CO_2_ reduction catalysts have shown improved stability and selectivity, they are usually tested under steady-state conditions or within narrow voltage–current ranges. PV-powered EC devices for the CO_2_RR offer high solar-to-chemical efficiency under fixed, standard lab conditions. However, the real PV output fluctuates with solar irradiance and temperature, requiring performance assessments under realistic, variable conditions. CO_2_RR catalysts must tolerate intermittent power while ensuring consistent product output across a broad dynamic range. This study demonstrates the viability of a silver-based gas diffusion electrode (Ag-GDE) for CO_2_ reduction in a directly coupled PV-EC system operating under realistic fluctuating solar conditions. The Ag-GDE, integrated into a full-flow EC cell with an iridium oxide anode, maintained stable CO selectivity and high energy coupling efficiency (0.96) across three day/night cycles emulated from the real Si-PV output. The system achieved cumulative solar-to-chemical efficiencies (STC_CO_) of 8.8, 8.2, and 7.8%, the highest reported for Si-PV-driven flow EC devices across a broad current density range (7–37 mA/cm^2^). This is the first demonstration of such performance under realistic dynamic conditions, highlighting the promise of Ag-GDE for efficient, stable, and scalable PV-powered CO_2_ conversion.

## Supplementary Material



## Notes

Effects of irradiance and temperature on PV current–voltage characteristics; PV-EC dynamic operation under realistic ambient conditions during a nonaccelerated day; PV-EC dynamic operation under realistic ambient conditions during a highly accelerated day; Ag-GDE characterization before and after the test; EDX maps of the post-test Ag-GDE cross section; electrochemical cell stability and frequency of electrolyte refill study; comparison of PV-EC operating in static and dynamic modes; preparation of silver gas diffusion electrode, CO_2_RR experiment, and PV-EC experiment with PV emulation; irradiance and temperature realistic ambient conditions; and PV-EC system efficiency evaluation (PDF)
