# Supplementary material for: Persistent CO2 Reduction Performance of an Ag Nanoparticle Gas Diffusion Electrode in Realistic Dynamic PV-Driven Operation
Source: Energy Fuels. 2025 Sep 24;40(1):811–8. doi: 10.1021/acs.energyfuels.5c03523 (PMC12797245; doi:10.1021/acs.energyfuels.5c03523)
Supplement: Supplementary file 1 [file ef5c03523_si_001.pdf]

Persistent CO_2 reduction performance of Ag nanoparticle gas diffusion electrode in realistic dynamic PV-driven operation

Thérèse Cibaka[a,d], Tsvetelina Merdzhanova[a], Oleksandr Astakhov[a], Sergey Shcherbachenko[a,c], Guangxin Liu[b], Chuyen van Pham[b], Uwe Rau[a,c], Peter Strasser[d]*

a. Forschungszentrum Jülich GmbH, IMD-3 Photovoltaik, Jülich 52428, Germany.

b. Helmholtz-Institute Erlangen-Nürnberg for Renewable Energy (IET-2), Forschungszentrum Jülich, Cauerstr. 1, Erlangen 91058, Germany.

c. Jülich Aachen Research Alliance (JARA-Energy) and Faculty of Electrical Engineering and Information Technology, RWTH Aachen University, Aachen 52062, Germany.

d. Technical University of Berlin, Institute of Chemistry, Berlin 10623, Germany.

Corresponding Author

* t.merdzhanova@fz-juelich.de

Experimental section

Preparation of Silver gas diffusion electrode

For catalyst ink preparation the silver catalyst powder (IoLiTec, 50–60 nm, 99.9 %) was first added to a mixture of nafion /Chemours, D2020), ethanol (Sigma Aldrich, AR) and water (MilliporeSigma, Mili-Q®, IQ7000, 18.2 MΩ.cm. The ink was dispersed and homogenized by a roller mixing (Ratek, BTR5) with a speed of 180rpm and 15g of Zirconium oxide grinding balls (Fritsh, 5 mm) [1]. The gas diffusion electrode (GDE) was fabricated by depositing the catalyst ink with a load of approximately 2 $mgcm^{-2}$ on cut GDLs (Freudenberg, H23C2) with a doctor blade (ZAA 2300, Zehnter) adjusted at a gap height of 300µm. The resulting gas diffusion electrodes (GDEs) were dried in a fume hood at room temperature in air atmosphere.

CO_2RR experiment

Electrochemical cell

CO_2 reduction reaction (CO_2RR) was performed in a commercially available microflow electrochemical cell (EC) from Electrocell. Figure M1 shows a schematic of the EC cell performing CO_2 reduction at the cathode (Silver-based gas diffusion electrode, Ag-GDE) and oxygen evolution at the anode (IrO_2^- coated titanium bulk plate provided by the EC manufacturer). The CO_2 flows through GDE and a CO_2-saturated 1M $KHCO_3$ electrolyte flows in and out of both the cathode and anode reaction chambers. The Ag-GDE cathode with a projected area of 9.5 cm^2 was prepared with homogenous coating and reaction site distribution enhancing CO selectivity. The cell was equipped with a Nafion 117 cation exchange membrane to separate cathode and anode compartments and to allow protons diffusion. CO_2 saturated 1mol/L $KHCO_3$ solution was used as both catholyte and anolyte. Catholyte and anolyte reservoirs were in-house built Teflon cup, with a volume capacity of 100 mL, equipped with liquid and gas inlet and outlet connections and a built-in temperature sensor. EC operations were maintained at 45 °C by heating all the electrolyte tubbings with heat pipes (HilleSheim, HT 60). During practical operation, particularly at high current densities,

unavoidable energy losses are converted into heat, causing the system temperature to rise to between 40 °C and 70 °C. While such elevated temperatures can enhance energy efficiency, they may also affect catalyst selectivity and compromise membrane stability [2]. Therefore, in this study, we maintained electrochemical cell (EC) operation at 45 °C to reflect practical operating conditions.

The continuous flow of catholyte and anolyte retard salt precipitation on the GDE or the membrane, while CO_2 purged in both catholyte, and anolyte assists the electrolyte pH balance. Every five hours, the electrolyte reservoirs were emptied and refilled with fresh 1M $KHCO_3$ to ensure ions balance and unchanged ionic conductivity. This time interval was determined with a

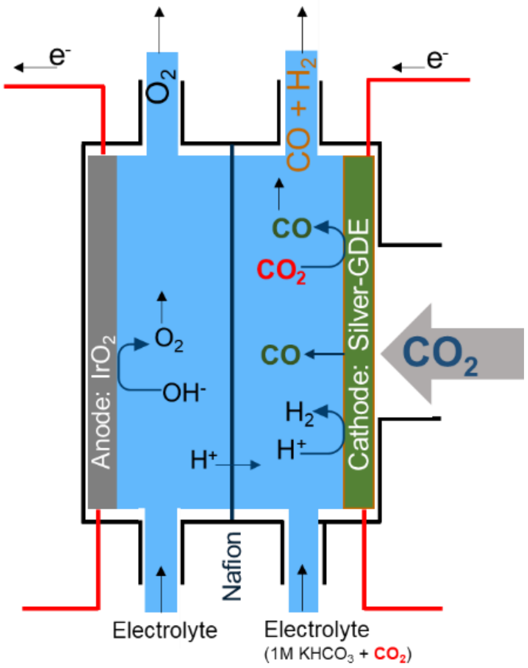

Figure M1. Schematic of electrochemical flow-cell operating with CO_2-saturated 1M $KHCO_3$ electrolyte performing CO_2 reduction at the cathode (Silver-GDE) compartment, Oxygen evolution at the anode (IrO_x). Nafion 117 membrane separates cathode and anode section.

periodicity study (available in supplementary information, Figure S10 where EC stability and electrolyte conductivity were performed during 20 hours with no electrolyte refreshment and during 15 hours with electrolyte refreshment every five hours. In this study, each EC compartment circulated

100 mL of electrolyte. For practical applications, increasing the electrolyte volume could help slow down the depletion of ionic conductivity during extended operation. Furthermore, the use of a bipolar membrane may help retain ionic species in both the catholyte and anolyte, contributing to improved ionic conductivity stability [3, 4].

The gas products mixed with unreacted CO_2 were analyzed by online gas chromatography (Shimadzu GC 30301) every 15min. GC was previously equipped with an in-built three-points calibration curve obtained with two-fold serial dilutions of a reference calibration gas (Linde and purity 5.6) consisted of 96% He, 1% H2, 1% CO, 1% CH_4 and 1% $CO_{2.}$ The gas dilutions were achieved by mixing the calibration gas with a pure He gas (Linde, 5.6).

CO_2 reduction setup

In the catholyte and anolyte reservoirs filled with 1M $KHCO_3$, CO_2 was constantly purged with a flow of 40 sccm. Using a peristaltic pump, the CO_2-saturated electrolyte was streamed from the reservoirs to the anode and cathode compartments of EC and back to the reservoirs with a 150mL/min flow rate. CO_2 was also purged directly inside EC through the gas diffusion electrode with a flow of 60 sccm. All gas flow rates were controlled by mass flow controllers. There was no dedicated gas outlet in the EC, all CO_2 and gas products streamed to the reservoir via the electrolyte.

The excess CO_2 and the gas products eluted in the catholyte reservoir and were analyzed by online gas chromatography (Shimadzu GC 30301) every 15min. GC was previously equipped with an in-built three-points calibration curve obtained with two-fold serial dilutions of a reference calibration gas (Linde and purity 5.6) consisted of 96% He, 1% H2, 1% CO, 1% CH_4 and 1% $CO_{2.}$ The gas dilutions were achieved by mixing the calibration gas with a pure He gas (Linde, 5.6). EC operations were maintained at 45 °C by heating all the electrolyte tubing with heat pipes (HilleSheim, HT 60).

PV-EC experiment with PV emulation

Under laboratory conditions, PV-EC experiments are usually carried out using a PV cell or module illuminated by a sun simulator under controlled temperature of 25°C. This setup construction, however, displays major limitations for studying PV-EC under various irradiance (G) and temperature (T) conditions over a long period of time [5]. The limitations are mostly due to insufficient irradiance and temperature flexibility and long-time irradiance and spectral stability. Another limitation is the size of the experimental EC cells, which typically have an area of approximately 1 cm^2 to 100 cm^2. Building a properly power-coupled PV-EC system of this size requires a downscaling of the PV devices to achieve proper ratio of PV-to-EC areas [6-11]. The corresponding downscaling of the PV modules in turn introduces additional constraints and losses. To overcome these limitations, we use a dedicated PV emulator to reproduce the IV characteristics of PV devices of the required type, size and number of cells at the target irradiance and temperature. The PV emulator built for the experiment allows to reproduce any PV current voltage characteristics with precision and accuracy on par with AAA class solar simulator [5] providing stability, reproducibility and flexibility required to bring an electrolyzer in the target PV application scenario.

In this work we emulated application of PV modules based on silicon heterojunction (SHJ) or Si-PV solar cells. The IV characteristics of a produced SHJ solar cell with an area of 246.21 cm^2 characterized under multiple combinations of (G, T) where *G* varied from 0.2 sun to 1.1 sun and *T* from 25°C to 60°C have been used to compose the time series of IVs representing the target "day". In order to match voltage and current of the emulated PV device to the characteristics of the EC cell, under static conditions at 1 sun 40°C and under dynamic conditions at 1 sun 53.3°C, we constructed the IV characteristics of a module with 5 cells connected in series and a total area of 44.6 cm^2 PV, this is represented in Figure M2 below. The number of cells allowed proper operating voltage range and the area of each cells ensure the corresponding operating current. Our PV module configuration displayed at 1.1 sun

a maximum power point with 3.2 V and 360 mA. In practical operation, with the utilization of electrochemical stacks, larger area of PV providing higher current are desired.

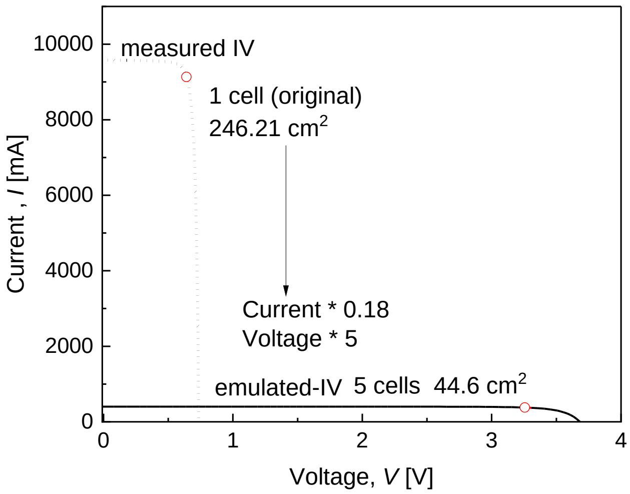

Figure M2. Current-voltage characteristic under standard test condition 1 sun A.M 15 at 25°C of the original large cell and the converted five-cells module. Maximum power point of each curve is the hollow red circle.

The PV-to-EC area ratio was 4.7. A schematic of the emulator connected with EC is shown in Figure M3. The connection between the SMU emulating PV device and EC cell had negligible ohmic losses.

PV emulation

The main goal of the study – test of CO_2 reduction in direct coupling to PV device under realistic conditions has been addressed with a specially designed PV emulator [14]. Utilizing the emulator exhibits three main benefits: resizing and reconfiguring an existing PV into any new desired PV without the necessity to physically modify the original PV, stable long-term PV operation avoiding typical sun simulator fluctuations due to lamps, rapid change of from one *G* and *T* conditions to the next one if needed.

The emulator is based on the algorithm that dynamically control output of a source measure unit (SMU) in such a way that the output reproduces an IV characteristic of any required PV device. The algorithm is realized as a Python script and in connection with Keithley 23060 SMU reproduces required original IVs with high accuracy. With this approach an accurate and reproducible experiment with a realistic time series of IVs was realized.

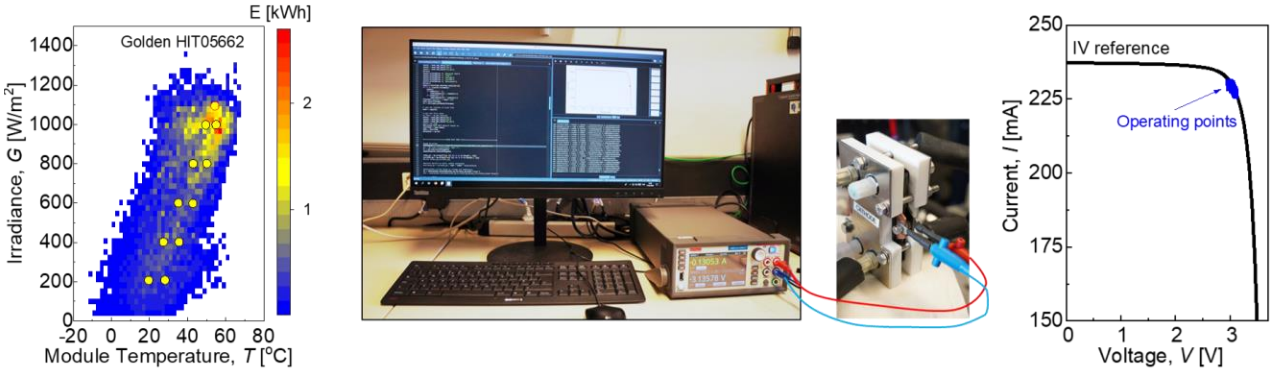

Figure M3. From left to right, Current-voltage characteristic of PV module emulated by a computer algorithm controlling an SMU connected to cathode and anode of EC. The graph on the right side shows the working or operating points of PV-EC device.

Irradiance and temperature realistic ambient conditions

To determine a minimal set of ambient conditions for our experiments we analyzed a year of measurements on a Silicon heterojunction PV module installed in Golden, Colorado – USA reported by NREL [12, 13]. The distribution of energy produced by the PV module during one year over temperature and irradiance is shown in Figure 1a in the Manuscript. A typical hypothetical summer day was simulated as presented in the table M1.

Table M1. Hypothetical summer day simulated in terms of irradiance and module temperature combinations

	Irradiance, sun	Temperature, °C
**Morning**	0.2	20
	0.4	27
	0.6	34
	0.8	42.8
**Noon**	1.0	49.8
	1.1	53.3
	1.0	53.3
**Afternoon**	0.8	49.8
	0.6	42.8
	0.4	35.8
	0.2	28.8

We designed a PV-module with IV characteristic providing optimal power coupling in PV-EC system at midday conditions of 1.1 sun-53.3°C. During PV-EC experiment under dynamic conditions, their operating current and voltage were recorded every 100 ms.

The daytime duration was condensed into a five-hours period. The experiment program consisted of three days lasting five-hour each followed by thirty minutes resting time (power off) representing nights. Total experiment duration was sixteen hours. After each day or 5-hours operation, the electrolyte inside EC device was refilled with fresh 1M $KHCO_3$ solution.

PV-EC system efficiency evaluation

PV-EC systems efficiency is ultimately evaluated by solar-to-chemicals (*STC*) efficiency, the fraction of the solar irradiance arriving at the PV device stored into the EC products. For the purpose of our study *STC* can be presented as a product of PV efficiency (η_{PV}), PV-EC coupling efficiency (C) and EC efficiency (η_{EC}) as formulated below. PV efficiency reflect how much of the sun power irradiated on the exposed area of the PV is converted in electric power at maximum power point:

$$\eta_{PV} = \frac{V_{MPP} I_{MPP}}{A_{PV} G} = \frac{P_{MPP}}{A_{PV} G} \quad (1),$$

where V_{MPP}, I_{MPP} and P_{MPP} are voltage, current and power of PV at the maximum power point, A_{PV} is the PV area and G is solar irradiance.

PV-EC power coupling efficiency represents how much of the maximum power deliverable by PV is actually being utilized by EC at operating point

$$C = \frac{V_{OP} I_{OP}}{V_{MPP} I_{MPP}} \quad (2),$$

Where V_{OP} and I_{OP} are voltage and current of EC at the operating point. To obtain operating current density J_{OP}, I_{OP} is divided by the area of EC, 9.5 cm^2.

PV-EC energy coupling efficiency represents how much of the maximum energy deliverable by PV is actually being utilized by EC during operating time.

$$C = \frac{\int V_{OP} I_{OP}}{\int V_{MPP} I_{MPP}} = \frac{E_{OP}}{E_{MPP}} \quad (3)$$

EC power efficiency towards one product (η_{ECp}) is the product of faradaic efficiency and voltage efficiency for the specific chemical. η_{ECp} reflects how much of the operating power (voltage and current) is actually used towards a specific chemical product (Eq. 4).

$$\eta_{ECp} = \frac{FE_p E_p^0}{V_{OP}} \quad (4),$$

where FE_p is the faradaic efficiency of a chemical product. CO_2 reduction to CO and HER are endothermic reactions, therefore Ep° is the thermoneutral voltage calculated with the enthalpy change for the specific product, E°, for CO is 1.47V and 1.47V for H_2.

Faradaic efficiency or selectivity towards a chemical product assess the utilization of the I_{OP} towards the formation of that specific chemical (H_2 or CO),

$$FE_p = \frac{n \times x_p \times F \times CO_2\ flowrate}{I_{OP}} \qquad (5),$$

Where n is the number of electrons required to obtain one molecule of the specific chemical (CO or H_2), x_p is the concentration of that chemical in ppm, F is the faradaic constant (96485 C/mol).

The overall EC efficiency η_{EC}, is the sum of all η_{ECp}.

$$\eta_{EC} = \sum \eta_{ECp} \qquad (6),$$

STC towards individual product is STC_p and *STC* towards all products.

$$STC_p = \eta_{PV}\ C\ \eta_{ECp} \qquad (7)$$

$$STC = \eta_{PV}\ C\ \eta_{EC} \qquad (8)$$

The EC cell used in our work produced CO and H_2 therefore we estimate *STC* for both of the products as well as the total *STC* as their sum.

Supplementary information

Effects of irradiance and temperature on PV current-voltage characteristics

The individual effects of temperature and irradiance on IV of PV module (5-cells, 51.7 cm^2) is depicted in Fig. S1. Generally, PV power generation is more greatly affected by irradiance than the temperature. Temperature influences mostly the voltage; for instance, at constant irradiance of 1 sun, the increase of temperature from 25 to 62°C resulted in constant I_{MPP} but a decrease of 0.37V in V_{MPP}. At 1 sun, P_{MPP} is 1228mW at 25°C and 1093 mW at 62°C. Parallelly, at constant temperature of 25°C, the irradiance variation from 1 sun to 0.2 sun, P_{MPP} decreased from 1093 mW to 217mW at 25°C, totaling decline of 82% of initial $P_{MPP.}$

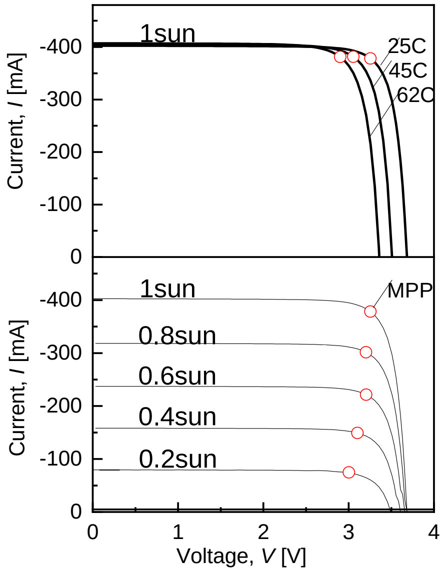

Figure S1. Current-voltage characteristics of the PV module. Top section: Constant irradiance of 1 sun and temperatures of 25°C, 45°C and 62°C. Down section: Constant temperature of 25°C and irradiances from 0.2 sun to 1 sun. Maximum power point of each curve is the hollow red circle.

Overall, photovoltaic systems perform optimally under high irradiance and lower temperatures. In real-world scenarios, high irradiance often leads to higher temperatures.

PV-EC dynamic operation under realistic ambient conditions during a non-accelerated day

Figure S2 displays PV-EC operating parameters, V_{OP}, I_{OP}, C, FE, during the non-accelerated day. As observed in the manuscript, both V_{OP}, I_{OP} followed irradiance pattern (symmetrical during the time frame of the experiment from 0.2 sun to 1.1sun to 0.2 sun). To eliminate possible effects of continuous modification of electrolyte ionic conductivity throughout long term experiment of 13 hours, we accelerated a simulated day time to five hours instead of thirteen in the manuscript.

In the non-accelerated and in the accelerated-day in the manuscript, the first hours of the single-day are characterized by very low faradaic efficiency towards H_2 despite low voltage. We believe that in both cases, Ag-GDE benefit from the high hydrophobicity of the fresh electrode. In the accelerated day (5 hours) presented in the manuscript, EC reaches again lower voltages near the fourth hour when simulating the evening and the FE_{H2} increases. In the non-accelerated day, EC voltages are higher around the fourth hours (near noon) and FE_{H2} is lower as expected. From the eleventh hour simulating the evening of the non-accelerated day, EC voltage is decreasing and FE_{H2} increases accordingly.

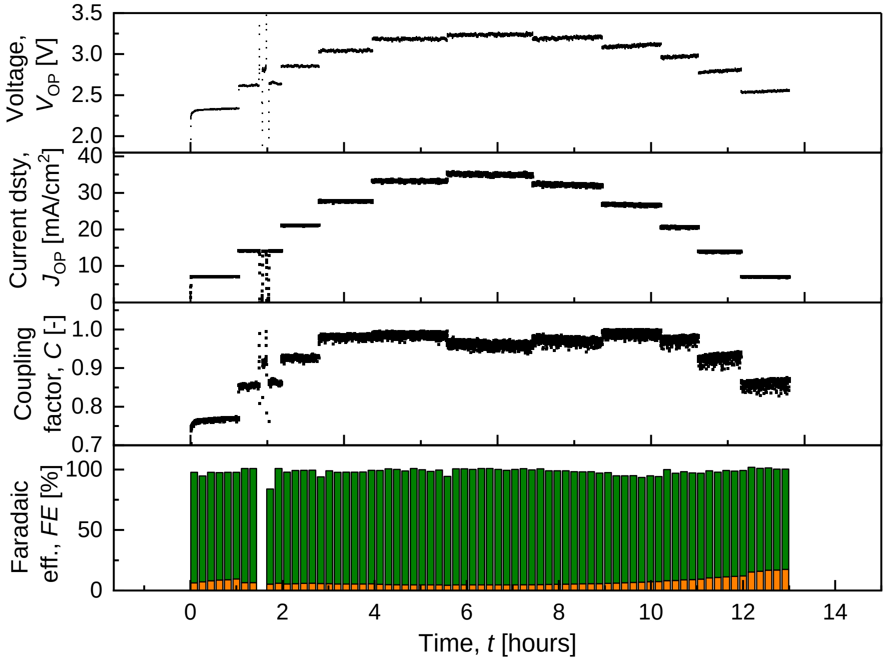

Figure S2. Operating voltage and current density, coupling factor of PV-EC and faradaic efficiencies at operating points during a simulated non-accelerated day.

In an additional experiment, we accelerated daytime even more and tested a succession of ten days lasting 20 minutes each with five minutes in between each day simulating nighttime. All cumulative ten days, presented in Figure S3, fitted in the time frame of five hours PV-EC operations. The measured gas products successfully showed a preference to CO over H_2 at either of these (G, T) conditions. The time frame of five hours (used in the manuscript) simulating a single day permit gas product identification at all *G* and *T* conditions.

Despite accurate observation of PV-EC operating current and voltage during this highly accelerated daytime, in the manuscript we maintained a time frame of 5 hours simulating day time for accuracy of solar-to-fuel efficiency.

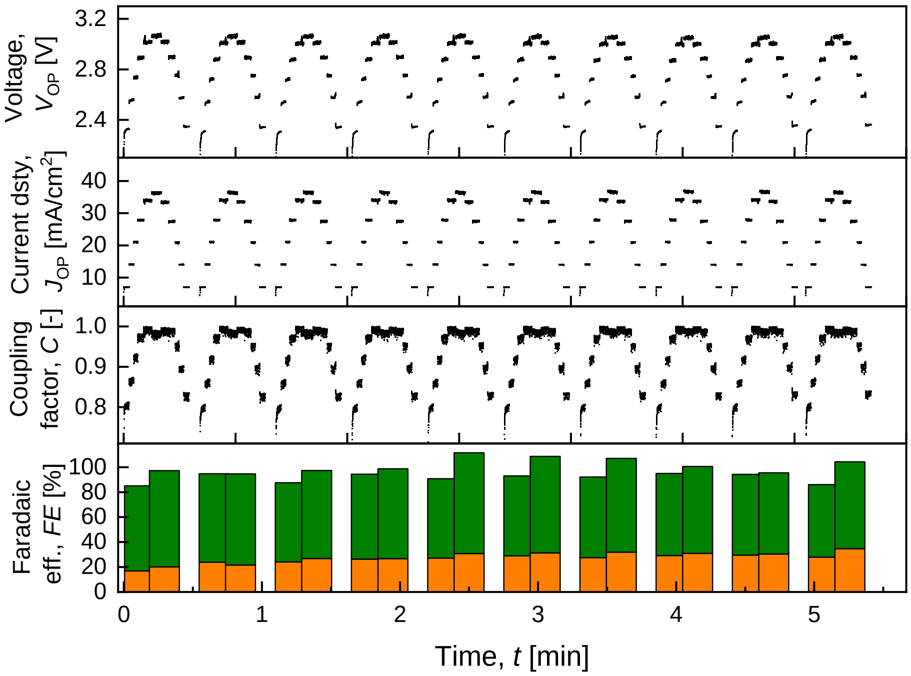

Figure S3. Operating voltage and current density, coupling factor of PV-EC and faradaic efficiencies at (0.4 sun, 28°C) and (1 sun, 49°C) during highly accelerated ten days lasting 20 minutes each.

Morphological structures of Fresh and used Ag-GDE

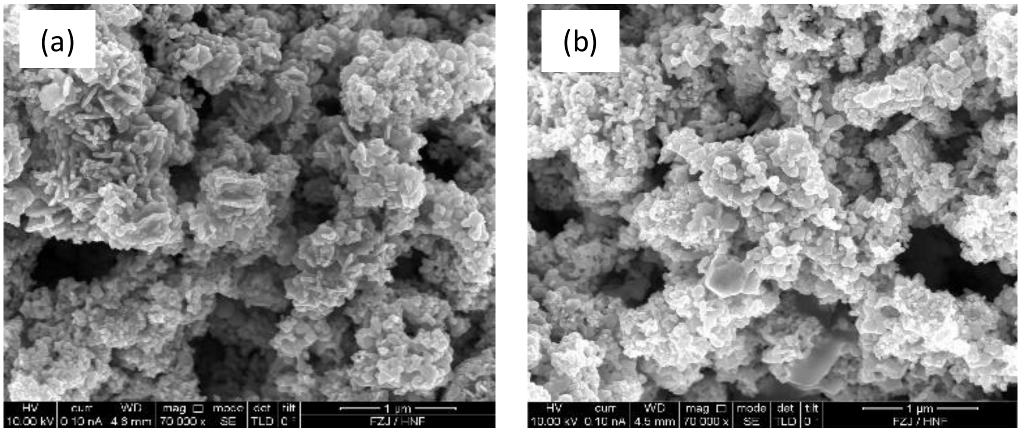

Figure S4. (a) SEM images of fresh Ag-GDE, (b) Used Ag-GDE displaying particles fused together.

Contact angles

Figure S5 presents the hydrophobicity decrease in terms of contact angle between a water droplet and the surface of Ag-GDE before any electrochemical test, a “one-day” old Ag-GDE and “three-days” old Ag-GDE.

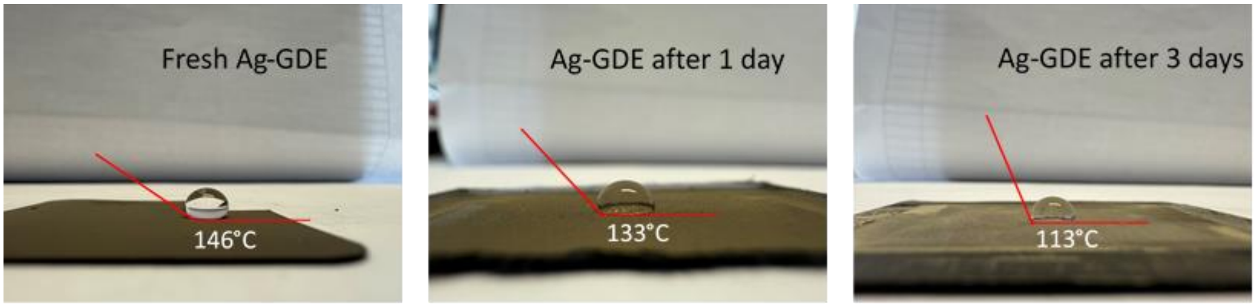

Figure S5. Evolution of hydrophobicity of a fresh, one-day operation old and three-days operation old Ag-GDE catalysts.

EDX maps of Ag-GDE post-test

After EC operation, the cross section of a used Ag-GDE was analyzed with SEM/EDX map for Ag, C, F and K, the images are displayed in Fig. S6. The silver in the upper layer is the catalyst layer, C (fibers and particles) is the main constituent of the GDL, Fluor derives from PTFE used by the GDL manufacturer as binding material for carbon fibers and carbon particles and K comes from residual electrolyte salt deposition in the Ag-GDE.

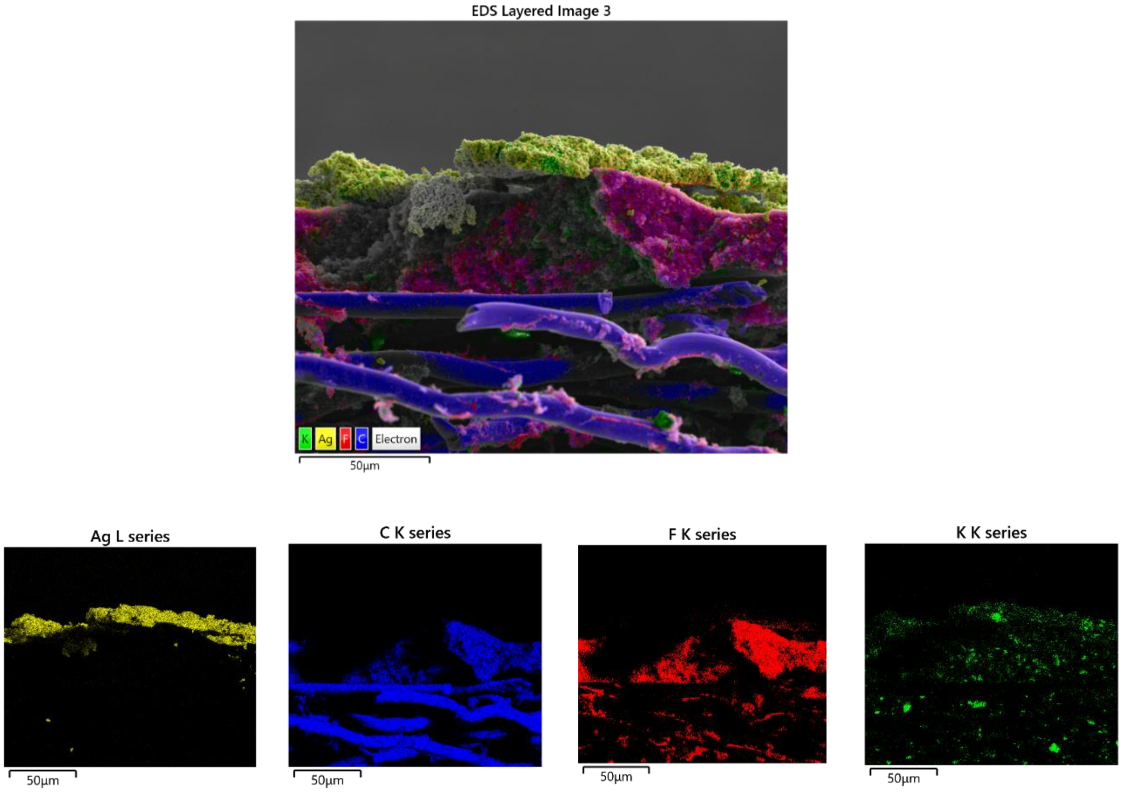

Figure S6. Cross section of a used Ag-GDE was analyzed with SEM/EDX map for Ag, C, F and K.

Electrochemical cell stability and frequency of electrolyte refill study

The temporal electric stability of EC was accessed via chronoamperometry (CA) at 3.0V (Fig. S7). In a first experiment CA of our EC lasted 18 hours, this is the black curve in Fig. S7. The current stayed relatively constant until the 5th hour before steadily decreasing until the 14th hour where the current reach nearly zero. For consistently steady operating current under a constant voltage, EC should maintain availability of active sites of electrodes, diffusion through membrane, ionic and electric conductivity, products and reactant transport. Diffusion through membrane can be hindered by electrolyte salt precipitation, this is partially avoided with continuous electrolyte flow. Ionic conductivity can be initially tuned with more concentrated electrolyte solution and maintained over time with electrolyte refreshment.

Products and reaction transport can be assured with CO_2 flow and electrolyte flow. To investigate whether the decrease of current was related to the electrodes change or electrolyte environment, we perform a second experiment in which the used electrolyte was emptied and replaced with a fresh 1M $KHCO_3$ solution every five hours.

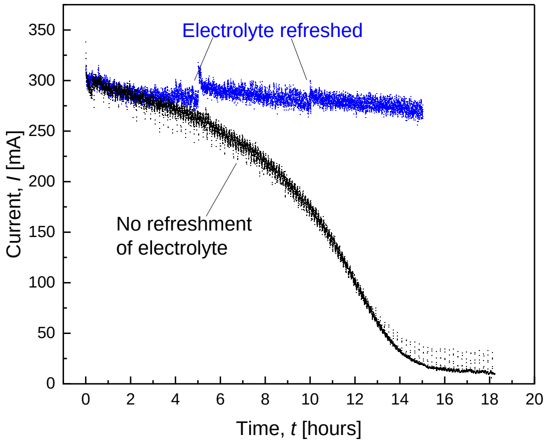

Figure S7. Temporal evolution of current at constant voltage cell of 3.0V during continuous 18 hours experiment and intermittent triple-five hours experiment.

The conductivities of the electrolytes were monitored in both experiments, these values are presented in table S1, after 18hours in the first experiment, the conductivity has decreased 100 times. The intermittent experiment has shown a stable current indicating that both cathode and anode are stable throughout the experiment but this stability is hidden in the first CA experiment by a change in the electrolyte conductivity. All voltammetry techniques were performed with OrigaLys OGF 05A potentiostat.

In order to maintain ionic conductivity, we periodically refreshed the electrolyte every five hours. The monitored ionic conductivities of the fresh electrolyte and electrolyte after 5 hours and 18 hours of EC experiment at constant cell voltage are shown in Table S1.

Table S1. Conductivities of fresh electrolyte, electrolyte after every 5-hours and electrolyte after 18 hours

Conductivity	Fresh electrolyte	After 5hours 1st section	After 5hours 2nd section	After 5hours 3nd section	After 18 hours
Catholyte	67 mS/cm	93 mS/cm	90 mS/cm	90 mS/cm	117 µS/cm
Anolyte		48 mS/cm	48 mS/cm	49 mS/cm	700 µS/cm

Constant voltage operation conditions

Using chronoamperometry at 3.0 V with a source measure unit, we simulated a PV-EC experiment under static operation conditions, maintaining constant 1 sun irradiance. The PV area (44.6cm^2) and efficiency were identical to those used in the dynamic PV-EC operation. In other words, constant irradiated sun power of 4460 mW on the exposed area of PV. The total cumulative charge passed under both static and dynamic conditions was the same: 2359.6 mA·h which is equivalent to two days operations under dynamic conditions and around 8 of hours operation under constant voltage of 3.0V and current of approx. 300 mA.

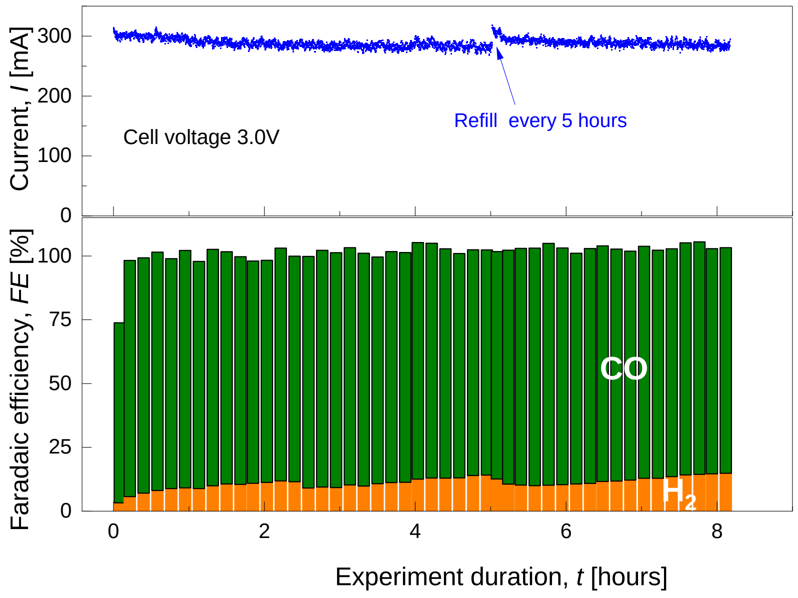

Figure S8. EC current and CO faradaic efficiency at constant 3.0V with electrolyte refreshment every 5 hours.

$$STC_{CO} = \frac{\int (I_{cell} \times FE_{CO} \times 1.47V)dt}{\int 4460\ mW\ dt}$$

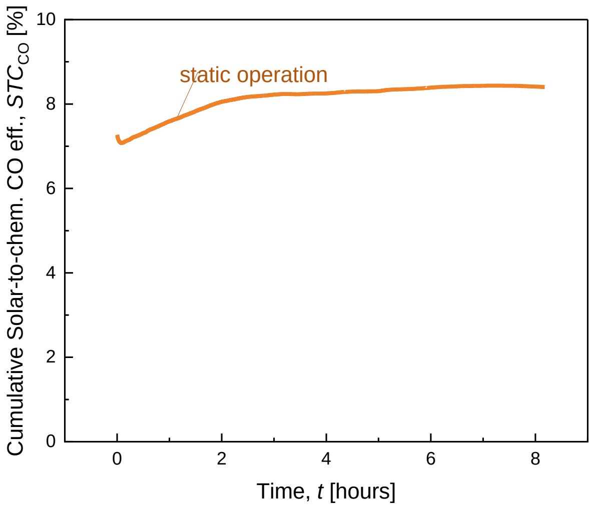

Figure S9. Evolution of STC_{CO} under simulated static conditions.

After one and two days of dynamic operation, STC_{CO} values decreased from 8.8% to 8.2%, respectively. In contrast, under charge-equivalent static conditions, the system maintained STC_{CO} values of 8.3% and 8.4%, supported by stable CO faradaic efficiency. On the first day of dynamic operation, the PV-EC system benefited from lower operating voltages at the beginning and end of the simulated day, combined with high CO selectivity throughout most of the day. However, on the second day, CO selectivity under dynamic conditions declined compared to day one.

EC operation under various cell potentials from 2.4V to 3.4V

Figure S10 below shows the static CO_2RR performance at potentials between 2.4 V and 3.4 V, evaluated in terms of faradaic efficiency. In this test, EC is power by a source measure unit, CO_2RR consistently exhibits high CO selectivity similar to the one when powered by PV.

In the experiment cell voltage was applied during 30 minutes with a resting time (power off) of 10 min, allowing EC to return to its initial state before operating under the next cell voltage.

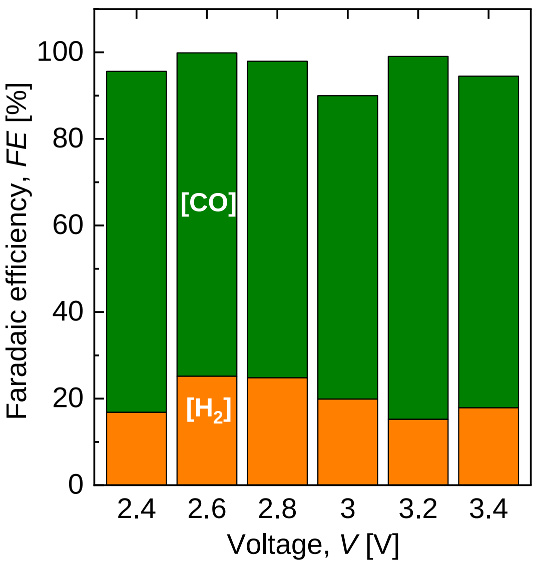

Figure S10. Static CO_2RR faradaic efficiency at 2.4–3.4 V, showing high CO selectivity comparable to PV operation.

Literature survey on STC_{CO} using PV-EC with various PV and EC technologies

Table S2. Summary of PV efficiency, EC efficiency towards CO, Solar to chemical efficiency towards CO and (G, T) operating conditions for different PV technology.

	η_{PV}, %	η_{EC^*} (CO), %	STC_{CO}, %	(G, T)	E° (CO) V	Ref.
Si-technology PV	20	35.9	6.5	1 sun, 40°C	1.34	[15]
	18	41.8	8.03	1 sun	1.34	[16]
	7.1	50.0	3.4	1 sun	1.34	[17]
	**21.41**	**41.3**	**8.8**	**1day (G, T)**	**1.47**	**Our**
	**21.41**	**38.4**	**7.86**	**3 days (G, T)**	**1.47**	**Work**

Thin film Silicon	8.2	34.8	2.85	1 sun	1.34	[18]
DSSC	2.61	33.5	0.79	1 sun	1.34	[19]
Perovskite PV	13.4	53.6	6.5	1 sun	1.34	[20]
III-V multijunction PV	28.5	45.0	13.4	1 sun, 25°C	1.34	[21]
	28.7	54.8	15.6	1 sun, 25°C	1.34	[22]
	29.3	73.9	15.9	1 sun	1.34	[23]
	29.3	81.4	18	1 sun	1.34	[23]
	28.5	51.6	13.9	1 sun	1.34	[24]
	31.6	50.5	15.5	1 sun	1.34	[25]
	32.7	59.5	19.1	1 sun	1.34	[26]

Unless stated in the article, $\eta_{EC^*\,(CO)}$, EC efficiency towards CO is calculated by the product of voltage efficiency ($E°_{CO}/V_{OP}$) and CO faradaic efficiency. In the main text, all STC_{CO} reported in the literature have been recalculated with thermoneutral potential of 1.47V.

References

1. Liu, G.X., et al., *Correlating catalyst ink design and catalyst layer fabrication with electrochemical CO2 reduction performance.* Chemical Engineering Journal, 2023. **460**.
2. Pelzer, H.M., et al., *Scaling and heating will drive low-temperature CO2 electrolysers to operate at higher temperatures.* Nature Energy, 2025. **10**(5): p. 549-556.
3. Mikami, N., et al., *Long Period Voltage Oscillations Associated with Reaction Changes between CO2 Reduction and H2 Formation in Zero-Gap-Type CO2 Electrochemical Reactor.* ACS Energy Letters, 2024. **9**(9): p. 4225-4232.
4. Gawel, A., et al., *Electrochemical CO2 reduction - The macroscopic world of electrode design, reactor concepts & economic aspects.* iScience, 2022. **25**(4): p. 104011.
5. M. F. Seidler, B.P., W. Zwaygardt, S. Haas, O. Astakhov, T. Merdzhanova, *A Photovoltaics Emulator for Electrochemistry Using Python and SCPI.* Journal of Power Sources, 2024. **under review**.
6. Sharifi, T., et al., *Toward a Low-Cost Artificial Leaf: Driving Carbon-Based and Bifunctional Catalyst Electrodes with Solution-Processed Perovskite Photovoltaics.* Advanced Energy Materials, 2016. **6**(20).
7. Atlam, O., F. Barbir, and D. Bezmalinovic, *A method for optimal sizing of an electrolyzer directly connected to a PV module.* International Journal of Hydrogen Energy, 2011. **36**(12): p. 7012-7018.
8. Sayedin, F., et al., *Optimal design and operation of a photovoltaic–electrolyser system using particle swarm optimisation.* International Journal of Sustainable Energy, 2014. **35**(6): p. 566-582.
9. Chang, W.J., et al., *Design Principle and Loss Engineering for Photovoltaic-Electrolysis Cell System.* ACS Omega, 2017. **2**(3): p. 1009-1018.
10. Clarke, R.E., et al., *Direct coupling of an electrolyser to a solar PV system for generating hydrogen.* International Journal of Hydrogen Energy, 2009. **34**(6): p. 2531-2542.
11. Sayedin, F., et al., *Optimization of Photovoltaic Electrolyzer Hybrid systems; taking into account the effect of climate conditions.* Energy Conversion and Management, 2016. **118**: p. 438-449.
12. W. Marion, A.A., C. Deline, S. Glick, M. Muller, G. Perrin, J. Rodriguez, S. Rummel, K. Terwilliger, T.J. Silverman, *User's Manual for Data for Validating Models for PV Module Performance*. 2014, National Renewable Energy Laboratory (NREL).
13. Marion, B., M.G. Deceglie, and T.J. Silverman, *Analysis of measured photovoltaic module performance for Florida, Oregon, and Colorado locations.* Solar Energy, 2014. **110**: p. 736-744.
14. Seidler, M.F., et al., *A photovoltaics emulator for electrochemistry using Python and SCPI.* Journal of Power Sources, 2025. **641**: p. 236723.
15. Sriramagiri, G.M., et al., *Toward a Practical Solar-Driven CO*

Flow Cell Electrolyzer: Design and Optimization. Acs Sustainable Chemistry & Engineering, 2017. **5**(11): p. 10959-10966.

16. Chae, S.Y., et al., *A perspective on practical solar to carbon monoxide production devices with economic evaluation.* Sustainable Energy & Fuels, 2020. **4**(1): p. 199-212.
17. Arai, T., et al., *Solar-driven CO2 to CO reduction utilizing H2O as an electron donor by earth-abundant Mn–bipyridine complex and Ni-modified Fe-oxyhydroxide catalysts activated in a single-compartment reactor.* Chemical Communications, 2019. **55**(2): p. 237-240.
18. Veenstra, F.L.P., et al., *CO(2) Electroreduction To Syngas With Tunable Composition In An Artificial Leaf.* ChemSusChem, 2024. **17**(4): p. e202301398.
19. Sacco, A., et al., *An Integrated Device for the Solar-Driven Electrochemical Conversion of CO2 to CO.* ACS Sustainable Chemistry & Engineering, 2020. **8**(20): p. 7563-7568.
20. Schreier, M., et al., *Efficient photosynthesis of carbon monoxide from CO2 using perovskite photovoltaics.* Nat Commun, 2015. **6**: p. 7326.

21. Schreier, M., et al., *Solar conversion of CO2 to CO using Earth-abundant electrocatalysts prepared by atomic layer modification of CuO.* Nature Energy, 2017. **2**(7): p. 17087.
22. Wang, Y., et al., *Efficient solar-driven electrocatalytic CO2 reduction in a redox-medium-assisted system.* Nature Communications, 2018. **9**(1): p. 5003.
23. Kim, B., et al., *Over a 15.9% Solar-to-CO Conversion from Dilute CO2 Streams Catalyzed by Gold Nanoclusters Exhibiting a High CO2 Binding Affinity.* ACS Energy Letters, 2020. **5**(3): p. 749-757.
24. Zhou, L.Q., et al., *A high-performance oxygen evolution catalyst in neutral-pH for sunlight-driven CO(2) reduction.* Nat Commun, 2019. **10**(1): p. 4081.
25. Mi, Y., et al., *Cobalt−Iron Oxide Nanosheets for High-Efficiency Solar-Driven CO2−H2O Coupling Electrocatalytic Reactions.* Advanced Functional Materials, 2020. **30**(31): p. 2003438.
26. Cheng, W.-H., et al., *CO2 Reduction to CO with 19% Efficiency in a Solar-Driven Gas Diffusion Electrode Flow Cell under Outdoor Solar Illumination.* ACS Energy Letters, 2020. **5**(2): p. 470-476.
